# A population approach to characterise amisulpride pharmacokinetics in older people and Alzheimer’s disease

**DOI:** 10.1007/s00213-016-4379-6

**Published:** 2016-08-01

**Authors:** Suzanne Reeves, Julie Bertrand, Fabrizia D’Antonio, Emma McLachlan, Akshay Nair, Stuart Brownings, Suki Greaves, Alan Smith, David Taylor, Robert Howard

**Affiliations:** 1 Division of Psychiatry, University College London, London, W1T7NF UK; 2 Department of Old Age Psychiatry, Kings College London, London, UK; 3 UMR 1137 IAME INSERM University Paris 7, France and Genetics Institute, University College London, London, UK; 4 South London and Maudsley NHS Foundation Trust, London, UK

**Keywords:** Amisulpride, Elderly, Alzheimer’s disease, Age, Population pharmacokinetics, Antipsychotic

## Abstract

**Introduction:**

Current prescribing guidelines for the antipsychotic amisulpride are based largely on pharmacokinetic (PK) studies in young adults, and there is a relative absence of data on older patients, who are at greatest risk of developing adverse events.

**Methods:**

This study aimed to develop a population PK model for amisulpride specifically in older people, by combining data from a richly sampled phase 1, single (50 mg) dose study in healthy older people (*n* = 20, 65–79 years), with a clinical dataset obtained during off label, low-dose (25–75 mg daily) amisulpride prescribing in older people with Alzheimer’s disease (AD) (*n* = 25, 69–92 years), as part of an observational study.

**Results:**

After introducing a scaling factor based on body weight, age accounted for 20 % of the inter-individual variability in drug clearance (CL), resulting in a 54 % difference in CL between those aged 65 and those aged 85 years, and higher blood concentrations in older patients.

**Discussion:**

These findings argue for the consideration of age and weight-based dose stratification to optimise amisulpride prescribing in older people, particularly in those aged 85 years and above.

**Electronic supplementary material:**

The online version of this article (doi:10.1007/s00213-016-4379-6) contains supplementary material, which is available to authorized users.

## Introduction

Safe and effective prescribing of antipsychotic medication is challenging in older people, as they are extremely susceptible to adverse events, including extrapyramidal side effects (EPS), falls, sedation and postural hypotension (Jeste et al. 2008; Leon et al. 2010). The mechanisms underpinning this heightened sensitivity are poorly understood (Uchida et al. 2009b), and research which aims to establish pharmacokinetic (PK) and pharmacodynamic (PD) contributions to clinical response and side effects in older clinical populations will be a key step towards improving safety profiles (Bigos et al. 2008a; Lotrich et al. 2006). This issue is particularly pertinent for older people with dementia, in whom excessive morbidity and increased cerebrovascular mortality have led to a restriction of antipsychotic use, and an emphasis on safety monitoring, but no clear guidance on minimum clinically effective doses when antipsychotic drugs are prescribed off licence to treat psychotic symptoms (Jennum et al. 2015; Maust et al. 2015).

The most widely held assumption is that age-related changes in peripheral pharmacokinetics, including changes in body composition, and a reduction in hepatic metabolism and renal clearance, lead to higher blood concentrations for a given drug dose (Mangoni and Jackson 2004; Merle et al. 2005). However, studies that have used a population approach (Duffull et al. 2011; Ette et al. 2004) to investigate PK profiles of antipsychotic drugs in older patients have shown that an age effect on drug clearance is not generalizable across antipsychotics (Bigos et al. 2008b; Feng et al. 2008), and emphasise the need to develop PK models for individual drugs.

Amisulpride is a second-generation antipsychotic drug widely used for the treatment of schizophrenia (Mauri et al. 2014), and for which optimal dose (400–800 mg daily), and therapeutic range of dopamine (D2/3) receptor occupancy (40–70 %), and blood concentration (100–319 ng/ml) have been clearly established (Hiemke et al. 2011; Lako et al. 2013; Sparshatt et al. 2009). These recommendations are based largely on patients below the age of 65 years, with minimal data on those aged 80 years and above. Pharmacokinetic studies of amisulpride (Coukell and Benfield 1996; Hamon-Vilcot et al. 1998; Rosenzweig et al. 2002) describe rapid absorption following oral administration, achieving peak plasma concentration (Cmax) after 1 h, and a second peak after 3 h, consistent with hepatobiliary elimination. The drug has low bioavailability (48 %) and low plasma protein binding (17 %) and is not a cytochrome P450 substrate. Amisulpride is eliminated unchanged in the urine (elimination half-life (*t*½) 12 h) and has high renal clearance (330 ml/min), suggestive of additional renal secretion (Dufour and Desanti 1988), possibly via the organic cation transport (OCT) system (Dos Santos Pereira et al. 2014; Jonker and Schinkel 2004). PK data on healthy older people are limited to a phase 1 study, which examined amisulpride PK characteristics in the first 72 h following a single (50 mg) dose (Hamon-Vilcot et al. 1998).

We have recently collected PK, [^18^F]fallypride D2/3 receptor imaging and clinical outcome data in older patients with AD who were prescribed amisulpride (25–75 mg daily) off label to treat psychotic symptoms, as part of an open observational study. To maximise the potential utility of this dataset to inform safer prescribing in AD, we aimed to use a population approach to establish the consistency and identify sources of variability in PK-PD relationships. This analysis represents the first stage of model development, with the following aims:

1. To develop a population PK model for amisulpride specifically for older people and AD, by combining the clinical dataset with published data from the single (50 mg) dose study
2. To investigate the contribution of physiological characteristics to inter-individual variability in PK parameters
3. To use model outputs to simulate and predict amisulpride dose-concentration relationships in people aged 65 years and over.

## Methods

### Data sources

#### Group 1

Twenty healthy elderly participants participated in a two-centre open study, which was approved by the Ethics Committee of the Pitie Salpetriere Hospital (Paris) (Hamon-Vilcot et al. 1998). Participants were included on the basis of having no haematological or biochemical abnormalities and were on no concomitant medication. Verbal and written informed consent was obtained prior to inclusion. Participants were sampled before and at 1, 2, 3, 4, 5, 6, 8, 10, 12, 24, 32, 48 and 72 h following a single tablet of 50 mg amisulpride, administered in the morning. Bloods concentrations of the amisulpride racemate were measured using a validated HPLC method based on liquid-liquid extraction and fluorescence detection. The method is linear from 0.5 ± 640 ng/ml in plasma, with a limit of quantification of 0.5 ng/ml. Both the racemate and enantiomers of amisulpride were measured, but only data on the racemate was used in the current analysis, for consistency with group 2 data.

#### Group 2

PK data were obtained from 25 patients with a diagnosis of probable AD (McKhann et al. 1984), who were participating in an observational study of amisulpride prescribing in older people. Patients were recruited from older adult mental health services based within the catchment area of the South London and Maudsley NHS Foundation Trust (SLaM) (London, UK), immediately prior to commencing low-dose amisulpride, which was being used off label to treat psychotic symptoms. Exclusion criteria included current or past history of psychiatric illness, being prescribed an antipsychotic or other oral drug that interferes with brain dopamine function, parkinsonian or other features suggestive of Lewy Body Dementia (McKeith et al. 1996), significant cardiorespiratory disease or needle phobia, and any contraindication to amisulpride use as stated in the summary of product characteristics (SmPc). Verbal and written informed consent was obtained from participants, or from a carer where a participant lacked capacity to give fully informed consent. The study was approved by the Berkshire Research Ethics Committee (REC reference 11/SC/0486). Clinical assessment was carried out at baseline and during dose titration: Psychotic symptoms and associated agitation were rated using the summed total score on three domains (delusions, hallucinations and agitation) of the carer-rated Neuropsychiatric Inventory (NPI) (Cummings et al. 1994), and extrapyramidal symptoms (EPS) were rated using the Simpson-Angus Scale (SAS) (Simpson and Angus 1970). Where possible, [^18^F]fallypride dopamine D2/3 receptor positron emission tomography (PET) imaging was carried out prior to amisulpride being commenced and when an optimum dose was achieved (25 % reduction in symptoms). Patients commenced amisulpride at a dose of 25 or 50 mg (based on the preference of the prescribing clinician), which was administered as a single evening dose, and increased to an optimum dose (25 % or greater reduction in symptoms and minimal EPS). Flexibility was built into the design around the timing of follow-up assessments, to account for variability in the dose titration regimen across prescribers and ensure that amisulpride concentration was obtained prior to each dose increase. As samples were taken at least 1 week after commencing amisulpride, all samples were assumed to be at steady state. In compliance with medication (pill counts and discussion with carer), changes in concomitant medication and clinical outcome (symptom ratings, side effects) were recorded at each visit. The timing of blood collection was not controlled and reflected convenience samples, which coincided with follow-up assessments and/or imaging. Date, time of sample and hours since last dose (confirmed by a carer where possible) were recorded on the anonymised assay request form. Blood samples were analysed in a secure, CPA-accredited laboratory (Clinical Toxicology Unit, Kings College Hospital). Amisulpride (racemate) blood concentrations were determined using validated liquid chromatography with tandem mass spectrometry (LC-MS/MS) method, with a detection limit of 9 ng/ml.

### Statistical analysis

Data were analysed using SPSS (version 22.0). Gender differences in demographic and physiological characteristics were explored using independent samples *t* tests. Correlations were expressed as Pearson’s correlation coefficient *r.*

### Population PK analysis

Nonlinear mixed effects modelling (NLME) was implemented using Monolix software (version 4.33; www.lixoft.eu). Diagnostic graphics and tests for covariate screening were performed in R. (version 3.2.4). Parameters were estimated using the Stochastic Approximation Expectation Minimisation (SAEM) algorithm (Kuhn and Lavielle 2005).

### Structural PK model

An oral two-compartment model was developed for amisulpride, with five parameters: (i) an absorption constant (ka), (ii) a central compartment (V1), representing blood and well-perfused tissues (e.g. liver, kidney), (iii) a peripheral compartment (V2) representing less well-perfused tissues (e.g. muscle, lean tissue, fat), (iv) an inter-compartmental distribution constant (Q), and (v) an elimination constant (CL), which includes renal and systemic clearance. The PK model was initially described using data from the single dose study (group 1), with initial estimates for the parameters being guided by the literature (Coukell and Benfield 1996; Hamon-Vilcot et al. 1998; Rosenzweig et al. 2002). Group 1 data was then combined with steady state data from AD patients (group 2). Blood concentration was converted from nanograms per millilitre to milligrams per litre for use in PK model building and concentrations below the limit of quantification (LOQ 5^e-4^ mg/l for group1 and 9^e-3^ mg/l for group 2) accounted for through simulation of the censored data with a truncated Gaussian distribution in the SAEM-Markov Chain Monte Carlo procedure (Samson and Lavielle 2006). Inter-individual variability (IIV) for PK parameters was estimated using an exponential model *P*_*i*_ = *P*_*TV*_ × *e*^*ηpi*^ where *P*_*i*_ and *η*_*pi*_ are the parameter estimate and corresponding random effect for the *i*th individual, and *P*_*TV*_ is the typical value for the parameter at the population level. The variability between the *i*th individual and population parameter values was described by *η*_*pi*_, which was assumed to be normally distributed with a mean of 0 and a variance of *ω*_*p*_^2^. In addition to IIV, a proportional residual error model (*y*_*ij*_ = *ŷ*_*ij*_ (1 + *ε*_*ij*_)) was used to describe random unexplained variability (system noise, dosage history errors and/or model misspecifications), where *y*_*ij*_ and *ŷ*_*ij*_ represent the *j*th *observed* amisulpride concentration of the *i*th subject, and its corresponding model *predicted* concentration, and *ε*_*ij*_ was assumed to be normally distributed with a mean of 0 and a variance of *σ*^2^. Residual error was modelled separately for each group, to account for inter-study differences.

### Covariate model

The contribution of physiological characteristics to IIV was assessed in a covariate screening which included weight, height, age and gender, and creatinine clearance (CrCL), which was estimated using the Cockcroft and Gault equation (Cockcroft and Gault 1976) and converted to l/h. Given the co-linearity between CrCL and other covariates of interest (weight, gender, age), serum creatinine was included as a separate covariate and allometric scaling for weight was introduced as an initial step. For group 1, clearance parameters (CL, Q) were set proportional to the body weight ratio over 70 kg to the power 0.75, and on the volume parameters (V1, V2) proportional to the body weight ratio over 70 kg to the power 1 (Mould et al. 2002). Covariates were then added in a stepwise procedure after visual inspection of covariate plots (continuous covariates were excluded if the correlation coefficient *r* between covariate and parameter was <0.25) and regression analysis in R. Continuous covariates which passed initial screening were introduced into the model after log transforming and centering on the mean. Gender was included as a categorical covariate. For the combined dataset, the absence of data on early time points meant that it was necessary to restrict allometric scaling for weight, and subsequent covariate testing, to CL.

### Model evaluation

Appropriateness of structural and covariate models was evaluated using goodness-of-fit criteria including diagnostic scatter plots, visual predictive checks, degree of shrinkage, change in IIV, model precision and likelihood ratio tests, with a 5 % threshold.

### Model predictions

Population estimates for CL (CL_TV_) and effect size of covariates (age and weight) on CL were used to estimate CL in an individual (CL_*i*_), for a given age and weight, using the equation:

$$ {CL}_{\mathrm{i}}={CL}_{TV}\ast {{\left({\mathrm{age}}_i/\mathrm{mean}\;\mathrm{age}\right)}^{\upbeta}}_{\mathrm{age},CL}\times {\left({\mathrm{weight}}_i/70\right)}^{0.75} $$

Terminal half-life (*t*½) was calculated as the ratio of log (2) and the second slope of elimination in the two-compartment model, itself derived from clearances and volumes population estimates.

### Model simulations

One hundred people were simulated for amisulpride concentration 15 h post-dose in each of the following categories: 65, 75 or 85 years; and of standard (70 kg) or low (50 kg) body weight, across the prescribed dose range (25, 50, 75 mg daily).

## Results

### Sample characteristics

#### Group 1

**Fig. 1:**
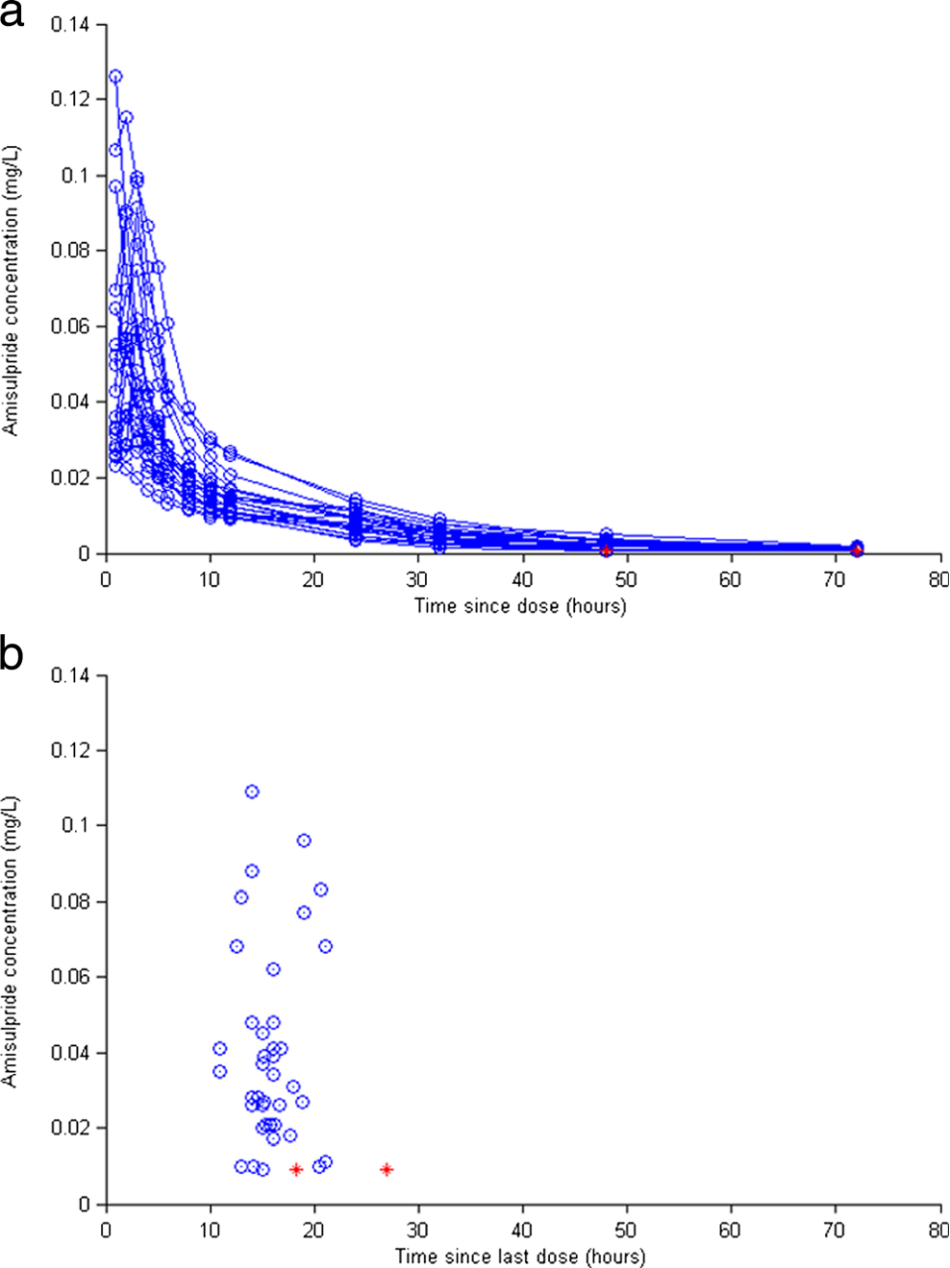
Scatterplot showing time since dose versus observed amisulpride concentration (mg/l) in group 1, following a single 50-mg oral dose (**a**), and group 2, at steady state across a dose range of 25–75 mg daily (**b**). Concentrations below the limit of the assay sensitivity are shown in *red* (colour figure online)

A total of 280 samples (14 per person) were obtained from 20 healthy elderly participants (10 men, mean age = 68.7 ± 4.1), over 72 h following a single oral 50-mg dose of amisulpride. Physiological characteristics of the sample are described in Table 1. Weight (*p* < 0.01), height (*p* < 0.01) and serum creatinine (*p* < 0.05) were significantly higher in men. CrCL was correlated with weight (*r* = 0.53, *p* < 0.05) and creatinine (*r* = −0.52, *p* < 0.05) but not age (*r* = −0.32, *p* = 0.16). A scatterplot of observed amisulpride blood concentration (mg/l) versus time (hours) since dose is shown in Fig. 1a. Cmax (64.0 ± 29.9 ng/ml) was observed after 2.1 ± 0.78 h. Cmax was higher in women (77.5 ± 28.8 ng/ml) than men (50.5 ± 25.4 ng/ml) (*p* < 0.05), but not correlated with age (*r* = −0.11, *p* = 0.64), height (*r* = −0.33, *p* = 0.15), weight (*r* = −0.33, *p* = 0.15) or CrCL (*r* = −0.17, *p* = 0.48).

**Table 1:** Physiological characteristics: group 1 (healthy elderly, single 50 mg dose)

Variable	Mean ± SD Total sample (*n* = 20)	Mean ± SD Men (*n* = 10)	Mean ± SD Women (*n* = 10)
Age (years)	68.7 ± 4.1	70.4 ± 5.1	67 ± 1.3
CrCL (ml/min)	80.5 ± 17.5	86.3 ± 18.2	74.4 ± 15.3
Weight (kg)	66.6 ± 9.1	73.7 ± 5.3 **	59.5 ± 6.1
BMI (kg/m^2^)	24.1 ± 2.4	25.2 ± 1.8 *	22.9 ± 2.5
Height (m)	1.7 ± 0.1	1.7 ± 0.5 **	1.61 ± 0.5
Creatinine (μmol/l)	56 ± 10.2	61.1 ± 9.7 *	51. 0 ± 8.4
Cmax (ng/ml)	64.0 ± 29.9	50.5 ± 25.4*	77.5 ± 28.8
Tmax (hours)	2.1 ± 0.8	2.4 ± 0.7	1.8 ± 0.8

Values for men and women were compared using independent samples *t* tests)

*CrCL* estimated creatinine clearance (ml/min—converted to l/h for the purposes of model building), *BMI* body mass index (kg/m^2^), *Cmax* peak plasma concentration (ng/ml—converted to mg/l for the purposes of model building), *Tmax* time taken to achieve Cmax (hours)

*p < 0.05; **p < 0.01

#### Group 2

A total of 41 samples (mean = 2 ± 0.78) were taken from 25 patients with AD (8(32 %) male; mean age = 82 ± 6.6 years mean Mini Mental State Examination (MMSE) = 18.4 ± 5.4) (Folstein et al. 1975), at 16.2 ± 3.1, over a dose range of 25–75 mg daily. All samples were taken at steady state and after 56.9 ± 58-day treatment. Physiological and clinical characteristics of AD patients are described in Table 2. Height (*p* < 0.05) and serum creatinine (*p* < 0.05) were higher in men. CrCL was significantly correlated with age (*r* = −0.65, *p* < 0.01), weight (*r* = 0.65, *p* < 0.01) and serum creatinine (*r* = −0.57, *p* < 0.01). A scatterplot showing observed amisulpride concentration (mg/l) versus time (h) since dose is shown in Fig. 1b. There was wide (>10-fold) variability in dose-corrected concentration (mean = 0.85 ± 0.53 ng/ml), which was correlated with age (*r* = 0.38, *p* = 0.01), but not weight (*r* = −0.04, *p* = 0.81), or CrCL (*r* = 0.05, *p* = 0.77), with no gender differences (*p* = 0.88).

**Table 2:** Sample characteristics: group 2 (Alzheimer’s disease, steady state amisulpride treatment)

Variable	Mean ± SD Total sample (*n* = 25)	Mean ± SD Men (*n* = 9)	Mean ± SD Women (*n* = 16)
Age (years)	81.8 ± 6.6	81.8 ± 7.7	81.8 ± 6.1
MMSE (maximum 30)	18.4 ± 5.4	19.8 ± 4.3	17.7 ± 5.8
CrCL (ml/min)	67.7 ± 17.3	67.8 ± 20.9	67.7 ± 17.4
Weight (kg)	68.0 ± 15.2	72.0 ± 8	65.8 ± 18
BMI (kg/m^2^)	26.5 ± 5.4	25.8 ± 1.8	26.9 ± 6.7
Height (m)	1.6 ± 0.1	1.7 ± 0.1*	1.6 ± 0.1
Creatinine (μmol/l)	83.1 ± 25.7	97.4 ± 31.3*	75.1 ± 18.5
Daily dose at time of sampling (mg)	49.4 ± 11.2	48.3 ± 6.4	48.1 ± 13.7
Time since dose (h)	16.2 ± 3.1	15.8 ± 3.2	16.3 ± 2.9
Days treatment, current dose	56.9 ± 58	62.6 ± 68.8	57.4 ± 56.8
Amisulpride concentration (ng/ml)	40.9 ± 27.1	41.5 ± 26.2	40.5 ± 28.1
Dose-corrected concentration (ng/ml/mg)	0.85 ± 0.53	0.87 ± 0.52	0.84 ± 0.54

Values for men and women were compared using independent samples *t* tests

*MMSE* Mini Mental State Examination, *CrCL* estimated creatinine clearance (ml/min—converted to l/h for the purposes of model building, *BMI* body mass index (kg/m^2^), *Cmax* peak plasma concentration (ng/ml—converted to l/h for the purposes of model building

*p < 0.05

### PK model

#### Group 1

**Fig. 2:**
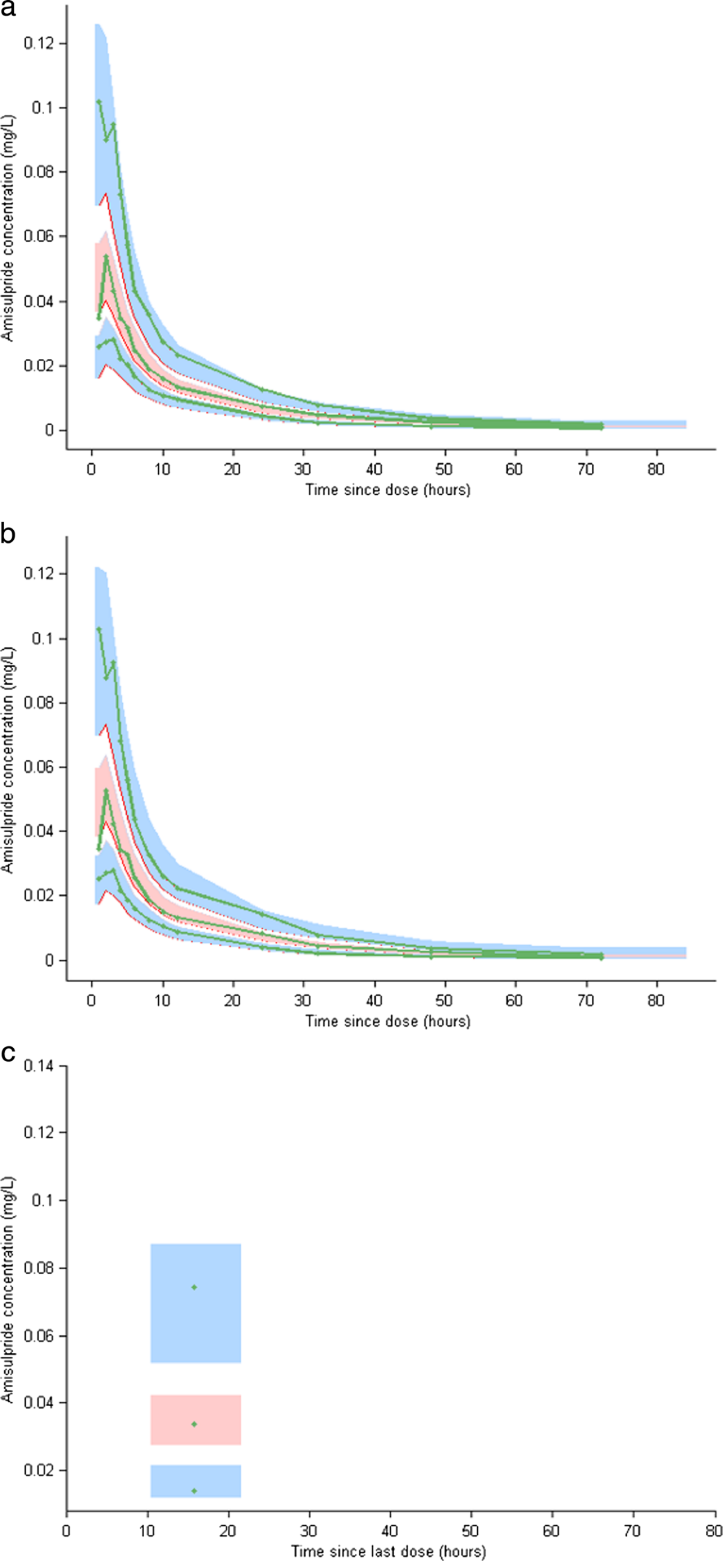
Visual predictive checks (VPC): 95 % prediction intervals around the 5th, 50th and 95th percentiles are shown for the final model after analysis of group 1 data alone, overlaid to group 1 observed 5th, 50th and 95th percentiles (**a**), and after analysis of the combined dataset overlaid to group 1 observed 5th, 50th and 95th percentiles (**b**), and overlaid to group 2 observed 5th, 50th and 95th percentiles (**c**)

An oral two-compartment model (parameterised in ka, CL, V1, Q, V2), which incorporated significant correlations for random effects between CL, Q, V1 and V2, was found to be the best fit for the data (see supplementary text and figures ESM_1, ESM_2). The model showed excellent precision, low (<5 %) shrinkage on clearance and volume parameters, and low (13 %) residual unexplained variability. Gender alone was a significant predictor of V1 (β_V1, gender = −0.52, *p* = 6.6^e-005^). Parameter estimates for the final model are summarised in Table 3, and visual predictive checks (VPC) shown in Fig. 2a. Population estimates for V1 were 40 % lower in women (399 l/h) than men (668 l/h), and estimated *t*½ (based on a 70 kg person) was 13.6 h for women and 15.2 h for men.

**Table 3:** Pharmacokinetic model estimates for group 1 and the combined (groups 1 and 2) sample

Parameter (units)	Group 1 (*n* = 20)		Combined (*n* = 45)	
	Estimate	RSE (%)	Estimate	RSE (%)
ka	0.87	14	0.85	16
Cl	84	7	54.3	8
β-_Cl,weight_	0.75	ne	0.75	ne
β-_Cl,Age_	ne	ne	−2.9 (*p* = 2.3^e-007^)	
V1	668 (Men)	15	455	13
	399 (Women)	13		
β-_V1, weight_	1	ne	ne	ne
β-_V1,Gender_	−0.52 (*p* = 6.6^e-005^)	25	ne	ne
Q	117	15	111	16
β-_Q,weight_	0.75	ne	ne	ne
V2 population	808	12	736	11
β-_V2,weight_	1	ne	ne	ne
Random effect				
ω_ka%	37	21	48	24
ω_Cl%	31	16	36	16
ω_V1%	42	21	43	27
ω_Q%	61	19	63	20
ω_V2%	50	17	46	18
ω_Cl_V1%	73	19	73	23
ω_Cl_Q%	68	21	60	29
ω_V1_Q%	51	48	73	23
ω_Cl_V2%	60	25	66	22
ω_V1_V2%	97	9	90	16
ω_Q_V2%	54	33	60	30
Residual error				
σ (group 1)%	13	5	13	6
σ (group 2)%	–	–	53	24

*ka* absorption constant, *Cl* apparent clearance from central compartment, *V1* central volume of distribution, *Q* intercompartmental clearance, *V2* peripheral volume of distribution, *ω* inter-individual variability (expressed as a percentage), *σ* residual unexplained variability (expressed as a percentage and separated on the basis of group), *weight* log transformed and centred around a standard 70 kg weight, *age* log transformed and centred around the mean, *RSE* relative standard error, *ne* not estimated

### Combined dataset

**Fig. 3:**
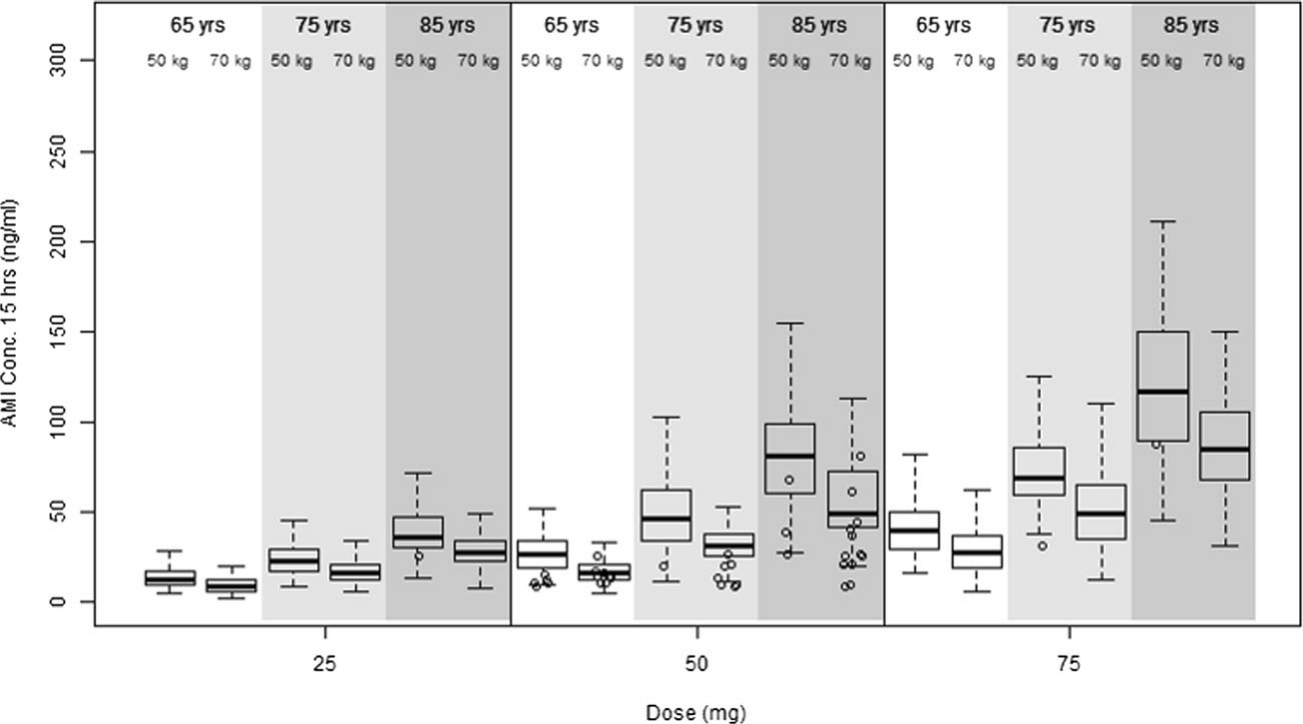
Simulated amisulpride concentrations (converted to ng/ml) at 15 h post-dose in a population of 100 people in each of the following categories: 65, 75 or 85 years old; and average (70 kg) or low (50 kg) body weight, across the prescribed dose range (25, 50, 75 mg daily). Observed data from the current study are represented as *circles*, binned by age (65 ± 5, 75 ± 5, 85 ± 5 years), weight (50 ± 10, 70 ± 10 kg) and dose

In the combined dataset, the population estimate for CL (54.3 l/h) was lower than that described for group 1 alone (84 l/h), and IIV on CL was higher (56 % in the combined, compared to 33 % in the group 1 dataset). Residual unexplained variability (RUV) was also higher for group 2 (53 %) than group 1 (13 %), and shrinkage was >45 % for all parameters apart from CL (27 %). Age was identified as the only significant contributor to IIV in CL (β_CL, age = −2.9, *p* = 2.3^e-7^). Parameter estimates for the best fit model are shown in Table 3, and VPCs are shown separately for group 1 (Fig. 2b) and group 2 (Fig. 2c). Based on population PK parameters (in a person of standard 70 kg weight), the impact of age was such that CL in those aged 85 years (41.8 l/h) was 54 % lower than those aged 65 years (91.1 l/h), and *t*½ extended from 12.4 to 22.7 h. Model simulations for amisulpride concentrations 15 h post-dose, accounting for age and body weight, are shown in Fig. 3.

## Discussion

We have developed a population PK model for amisulpride in the elderly by combining data on healthy older people with a representative sample of AD patients, who were receiving low-dose amisulpride off label specifically to treat psychotic symptoms. The fact that PK data in group 1 was collected at multiple time points meant that it was possible to fully parameterise an oral two-compartment PK model and carry out covariate testing on all clearance and volume parameters. The final model explained 87 % of the variance in PK profile with excellent precision and identified gender as a significant contributor to the variability in central volume of distribution (V1). Gender differences in V1, which led to a higher Cmax in women and longer estimated *t*½ in men (15.3 h compared to 13.6 h in women), were not explained by differences in body mass, as weight was accounted for in the model through the use of allometric scaling, and height was compared against gender as part of covariate screening and model development.

The addition of group 2 data increased the estimated inter-individual variability in drug clearance from 31 % (group 1 data alone) to 56 % (combined sample). This variability was accounted for by a significant effect of age on drug clearance, consistent with but not solely accounted for an age-related reduction in CrCL. The magnitude of the effect was such that CL at 85 years was 54 % lower than at 65 years, resulting in increased concentrations for a given dose in older patients. Whether the observed age effect represents primarily an effect of age on excretion, secretion or possibly both is unclear, but this warrants further investigation, as it has implications for the prescribing of other OCT substrates, such as clozapine (Haenisch et al. 2012), in older patients. It will also be important to further explore the nature of the age effect on CL, as it is likely that the effect of age increases exponentially across the age spectrum, resulting in changes in CL of a higher order of magnitude in the oldest individuals.

Introducing allometric scaling into the covariate model (Holford 1996; Mould et al. 2002) allowed CL to be estimated across the wide range of observed weights in the clinical dataset. Model simulations suggest that steady state blood concentrations at 50 mg amisulpride daily would increase from 30 to 85 ng/ml between the ages of 65 and 85 years in a 70-kg person, and from 40 to 120 ng/ml in a person of low (50 kg) body weight. These findings argue for the consideration of age- and weight-based dose adjustments in older people. Previously conducted clinical studies have shown an increase in CL and extended *t*½ in patients with renal impairment (Rosenzweig et al. 2002). As a result, a 50 % reduction in daily dose is advised in moderate renal disease (GFR 30–60 ml/min) and a 66 % reduction in severe renal disease (GFR is 10–30 ml/min). The PK model developed in this analysis included eight AD patients with moderate renal disease, a common finding in older patients, and one advantage of building the model from a representative clinical dataset. Our findings suggest that, for patients aged 65 years and above who do not have severe renal impairment, age and weight could be considered in place of renal status, to guide dose reductions.

The published literature on amisulpride (Mauri et al. 2014; Sparshatt et al. 2009) has consistently reported higher dose-corrected concentrations in women during TDM, and it has been assumed that gender differences in drug clearance contribute to this (Coukell and Benfield 1996). However, data from the four large, naturalistic studies which have contributed most to our understanding of amisulpride PK are comprised predominantly of patients below 65 years and include minimal data on patients aged 80 years and above (Bergemann et al. 2004; Muller et al. 2009, 2007, 2006). In the current study, there were no gender differences in dose-corrected concentrations at steady state, and neither was there an impact of gender on the variability in CL. Our findings suggest that, after the age of 65 years, no additional dose adjustments are required on the basis of gender, after accounting for age and body weight.

There are several limitations to the analysis, including the relatively small sample size and sparse sampling in the clinical study. This meant that it was not possible to estimate within-subject (inter-occasion) variability in CL, which reduced the predictive power of the model and contributed to the high residual unexplained variability (53 %). It was beyond the scope of the current analysis to examine the contribution of other covariates such as concomitant medication, as the study was not powered to incorporate multiple categorical variables into the covariate model. This is however balanced by the fact that amisulpride is not a CYP450 substrate, which reduces the likelihood of potential drug interactions, and patients who were being prescribed drugs known to interact with amisulpride, including OCT substrates lithium and clozapine (Bowskill et al. 2012), were excluded from participation in the study. We cannot completely rule out the possibility that changes in renal function through the course of treatment contributed to the observed variability in CL, as additional monitoring of renal function was not routinely carried out during dose titration, and there was wide variability in length of follow-up due to technical difficulties around the imaging procedure. Compliance is another important source of potential variability in blood concentration and particularly relevant in cognitively impaired patients. As a result, compliance was carefully monitored (caregiver report and tablet counts) and facilitated by the clinical team.

Despite the limitations of the AD dataset, it represents the first PK data on amisulpride within the context of therapeutic, steady state dosing in a clinical population who reached the extreme end of the age spectrum. The magnitude of the change indicates that therapeutic blood concentrations are achieved at lower doses and that age and body weight could be considered in place of renal status to guide dose adjustments. Obtaining additional steady state data on elderly people will help to further refine the model predictions regarding gender, concomitant medications and co-morbid health conditions, including pre-existing renal impairment.

Previous studies which have used a population approach to explore PK profiles of psychotropic drugs in older AD patients have produced mixed findings. For example, PK models developed using data from the Clinical Antipsychotic Trials of Intervention Effectiveness (CATIE) trials for Alzheimer’s disease (AD) and schizophrenia (SZ) have shown a significant effect of age on clearance of the active metabolite (9-OH risperidone) of risperidone (Feng et al. 2008), whereas inter-individual variability in olanzapine clearance was accounted for by factors other than age (gender, smoking and African-American race) (Bigos et al. 2008b). These data, and a recent publication from the Citalopram in Alzheimer’s disease (CitAD) study, which showed significant and clinically relevant effects of age and gender on metabolic clearance of R- but not S-citalopram (Akil et al. 2016), serve to emphasise the importance of extending pharmacological modelling to representative older clinical populations, to meaningfully refine and optimise age- and disease-specific dose adjustments.

The very low doses of amisulpride prescribed to AD patients in this study are considerably lower than those used to treat psychotic symptoms in schizophrenia (Hiemke et al. 2011; Sparshatt et al. 2009) and at the lower end of the suggested efficacious dose range from open label studies which have similarly prescribed amisulpride off licence to treat patients with AD and psychotic symptoms (50–200 mg) (Lim et al. 2006; Mauri et al. 2006). This reflects the primary aim of the study, which was to establish the minimum clinically effective dose required to reduce symptoms without EPS. As a result, dose titration regimens commenced at doses as low as 25 mg (half a tablet) and increased to an optimum, based on response and side effect profile. Of the 25 patients included in the study, only one achieved a blood concentration (109 ng/ml) within the therapeutic range recommended for the treatment of positive symptoms in schizophrenia (Hiemke et al. 2011; Sparshatt et al. 2009). This suggests that either patient were sub-optimally treated in the current study or that the target therapeutic range is lower in older AD patients, as a result of age- and/or disease-related changes in central pharmacokinetics (Clark-Papasavas et al. 2014; Seeman 2014) or altered pharmacodynamics (neurotransmitter, receptor or signal transduction level), which lower the therapeutic D2/3 occupancy range (Graff-Guerrero et al. 2015; Uchida et al. 2009a). This will be investigated in future analyses, which will aim to combine the PK model with data on D2/3 receptor imaging and clinical outcome, to explore PK occupancy profiles, establish the target concentration and D2/3 occupancy range to avoid non-response and EPS, and further inform AD-specific dose adjustments.

## Electronic supplementary material

ESM_1 (JPEG 55 kb)

High resolution image (TIFF 86 kb)

ESM_2 (JPEG 46 kb)

High resolution image (TIFF 280 kb)
