# Supplementary figures and images for: A population approach to characterise amisulpride pharmacokinetics in older people and Alzheimer’s disease
Source: Psychopharmacology (Berl). 2016 Aug 1;233:3371–81. doi: 10.1007/s00213-016-4379-6 (PMC4989015; doi:10.1007/s00213-016-4379-6)

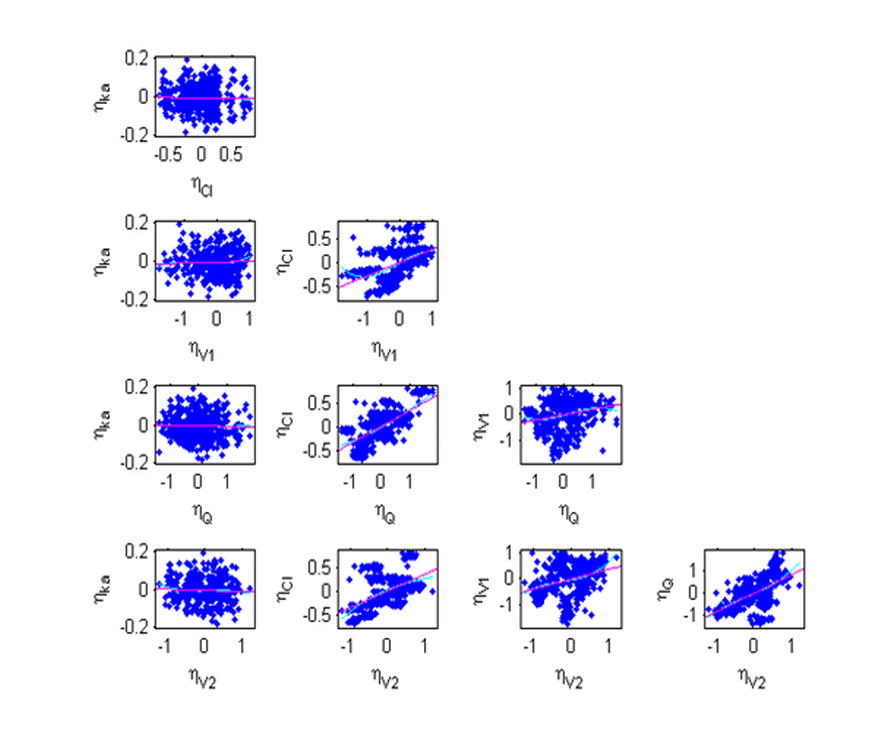

Supplement: Supplementary file 1 — (JPEG 55 kb) [file 213_2016_4379_Fig4_ESM.jpg]

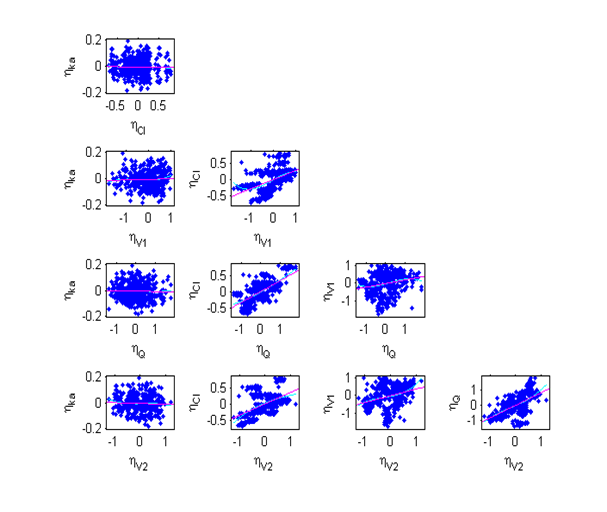

Supplement: Supplementary file 2 — High resolution image (TIFF 86 kb) [file 213_2016_4379_MOESM1_ESM.tif]

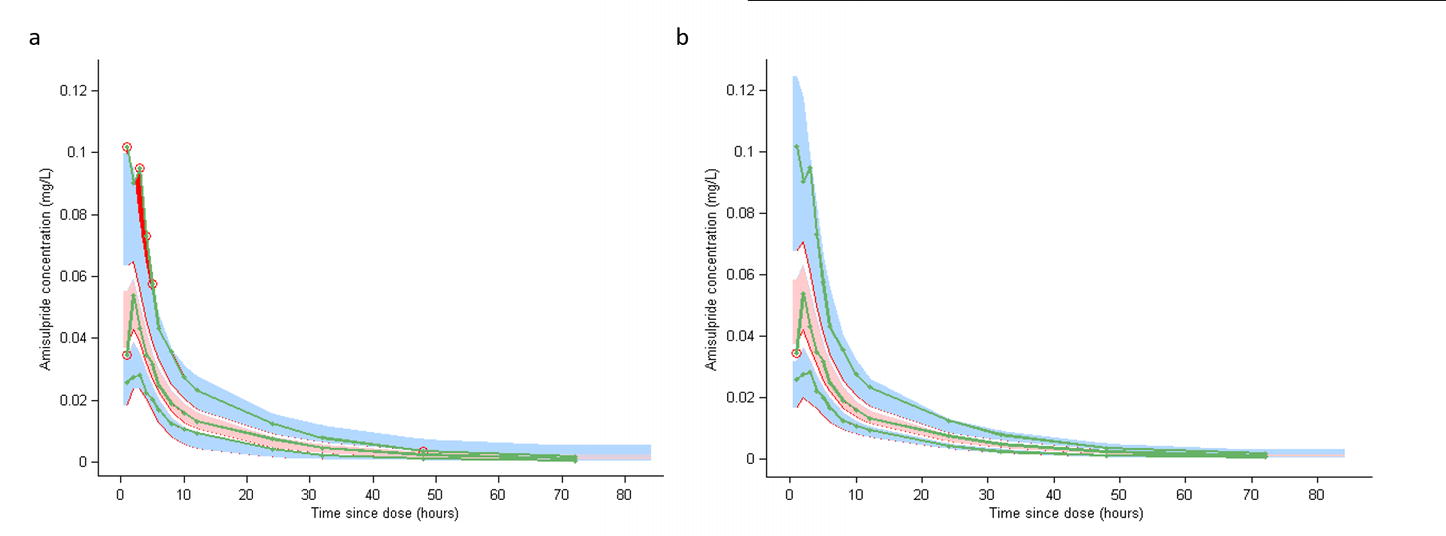

Supplement: Supplementary file 3 — (JPEG 46 kb) [file 213_2016_4379_Fig5_ESM.jpg]

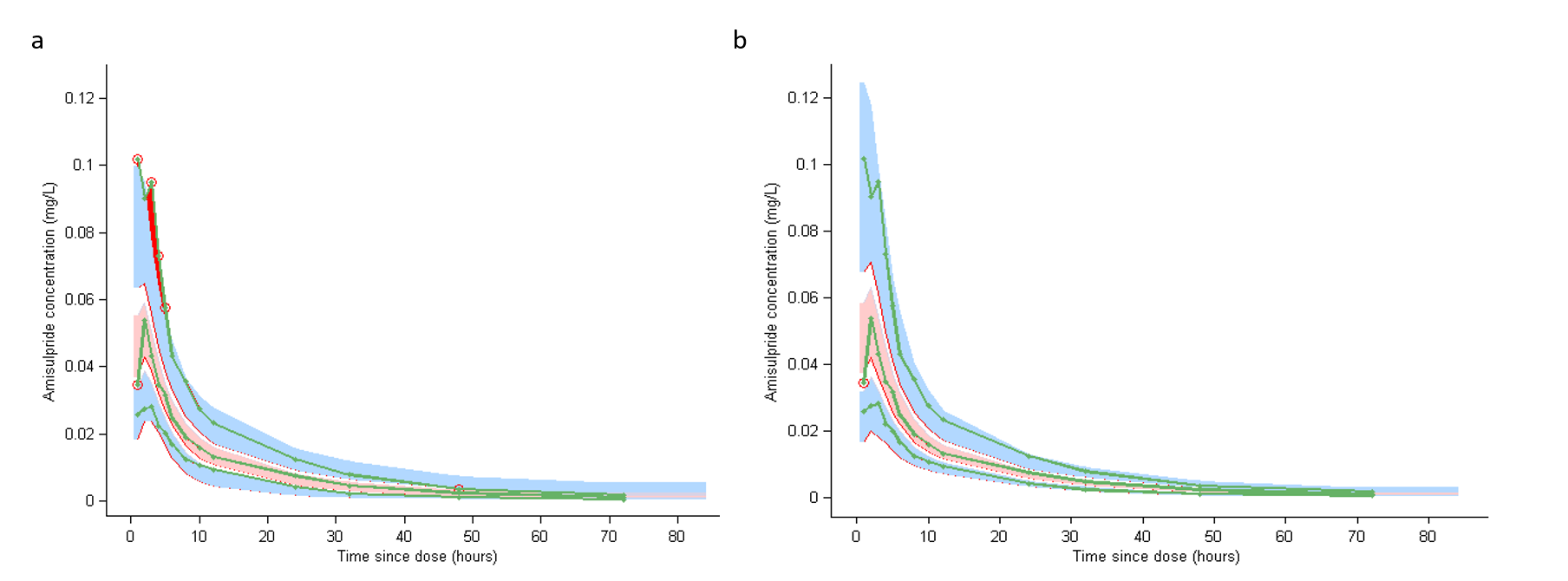

Supplement: Supplementary file 4 — High resolution image (TIFF 280 kb) [file 213_2016_4379_MOESM2_ESM.tif]
